# Prospective association between handgrip strength and cardiac structure and function in UK adults

**DOI:** 10.1371/journal.pone.0193124

**Published:** 2018-03-14

**Authors:** Sebastian E. Beyer, Mihir M. Sanghvi, Nay Aung, Alice Hosking, Jackie A. Cooper, José Miguel Paiva, Aaron M. Lee, Kenneth Fung, Elena Lukaschuk, Valentina Carapella, Murray A. Mittleman, Soren Brage, Stefan K. Piechnik, Stefan Neubauer, Steffen E. Petersen

**Affiliations:** 1 Department of Epidemiology, Harvard T.H. Chan School of Public Health, Boston, Massachusetts, United States of America; 2 William Harvey Research Institute, NIHR Biomedical Research Center at Barts, Queen Mary University of London, London, United Kingdom; 3 Division of Cardiovascular Medicine, Radcliffe Department of Medicine, University of Oxford, West Wing, John Radcliffe Hospital, Headington, Oxford, United Kingdom; 4 Cardiovascular Epidemiology Research Unit, Cardiovascular Division, Beth Israel Deaconess Medical Center, Harvard Medical School, Boston, Massachusetts, United States of America; 5 MRC Epidemiology Unit, University of Cambridge, Cambridge, United Kingdom; Universita degli Studi di Napoli Federico II, ITALY

## Abstract

**Background:**

Handgrip strength, a measure of muscular fitness, is associated with cardiovascular (CV) events and CV mortality but its association with cardiac structure and function is unknown. The goal of this study was to determine if handgrip strength is associated with changes in cardiac structure and function in UK adults.

**Methods and results:**

Left ventricular (LV) ejection fraction (EF), end-diastolic volume (EDV), end-systolic volume (ESV), stroke volume (SV), mass (M), and mass-to-volume ratio (MVR) were measured in a sample of 4,654 participants of the UK Biobank Study 6.3 ± 1 years after baseline using cardiovascular magnetic resonance (CMR). Handgrip strength was measured at baseline and at the imaging follow-up examination. We determined the association between handgrip strength at baseline as well as its change over time and each of the cardiac outcome parameters. After adjustment, higher level of handgrip strength at baseline was associated with higher LVEDV (difference per SD increase in handgrip strength: 1.3ml, 95% CI 0.1–2.4; p = 0.034), higher LVSV (1.0ml, 0.3–1.8; p = 0.006), lower LVM (-1.0g, -1.8 –-0.3; p = 0.007), and lower LVMVR (-0.013g/ml, -0.018 –-0.007; p<0.001). The association between handgrip strength and LVEDV and LVSV was strongest among younger individuals, while the association with LVM and LVMVR was strongest among older individuals.

**Conclusions:**

Better handgrip strength was associated with cardiac structure and function in a pattern indicative of less cardiac hypertrophy and remodeling. These characteristics are known to be associated with a lower risk of cardiovascular events.

## Introduction

Cardiovascular disease (CVD) accounts for 17.3 million deaths per year worldwide and is expected to account for 23.6 million by 2030 [1]. It is, therefore, important to identify predictors of CVD incidence to be able to initiate evidence-based primary prevention among individuals at elevated risk. Handgrip strength is an inexpensive, reproducible and easy to implement measure of muscular fitness that has been repeatedly shown to be associated with CVD incidence [2–7], independent of measures of body composition such as muscle area and BMI [2].

The association between handgrip strength and CVD has been demonstrated in various settings. Sasaki et al [4] showed that the strength of the association between handgrip strength and cardiovascular mortality is similar among men and women and Ortega et al [7] showed an association between handgrip strength and premature cardiovascular death among adolescents. More recently, the Prospective Urban Rural Epidemiology (PURE) study demonstrated that the relationship between grip strength and CVD is consistent across a wide range of country-specific incomes [3]. Further study is needed to investigate potential underlying pathophysiologic mechanisms linking handgrip strength to CVD incidence.

Recently, several pathways have been proposed through which sarcopenia, a cause of low handgrip strength, could contribute to heart failure with preserved ejection fraction [8]. Among those are activation of systemic inflammation [9] and insulin resistance [10]. Cardiac changes seen in patients with heart failure with preserved ejection fraction include concentric left ventricular remodeling and concentric hypertrophy [11]. Thus, the observed association between handgrip strength and CVD incidence may be due to less cardiac remodeling and hypertrophy among individuals with better handgrip strength. The use of cardiac magnetic resonance (CMR) imaging is the reference standard to accurately determine cardiac structure and function [12]. However, no studies exist that have described the relationship with handgrip strength.

The goal of this study is to investigate the association between muscular fitness as repeatedly assessed by handgrip strength and cardiac structure and function as measured by CMR in a large sample of UK adults.

## Methods

### UK Biobank

The UK Biobank (http://www.ukbiobank.ac.uk) is a prospective cohort study of more than 500,000 men and women aged 40–69 at the time of recruitment between 2006 and 2010 in 22 centers across the UK. The baseline assessment of study participants included an extensive questionnaire, a physical assessment including height, weight, body fat, blood pressure, pulse rate, and handgrip strength, and collection of biological samples. Follow-up of participants was conducted via linkage to health record systems and re-contact with the participants. The study complies with the Declaration of Helsinki. All participants provided written consent and UK Biobank’s scientific protocol and operational procedures were reviewed and approved by the North West Research Ethics Committee in the UK.

### Exclusion criteria

We excluded participants with any history of cardiovascular conditions Details are available in the supporting information (S1 Methods).

### Cardiovascular magnetic resonance imaging

The UK Biobank invited participants back for a comprehensive imaging visit [13, 14] including a 20-minute CMR examination at 1.5 Tesla with a goal to perform 100,000 CMR scans. The current study represents an interim data release with 5,065 participants.

The CMR protocol and image analysis have been previously described [14]. In brief, CMR imaging is being performed in Cheadle, United Kingdom, on a clinical wide bore 1.5 Tesla scanner (MAGNETOM Aera, Syngo Platform VD13A, Siemens Healthcare, Erlangen, Germany). 18 channels anterior body surface coil was used in combination with a 12 elements of an integrated 32 element spine coil and electrocardiogram (ECG) gating for cardiac synchronization. Acquisitions include piloting and sagittal, transverse and coronal partial coverage of the chest and abdomen. For cardiac function, three long axis cines (horizontal long axis—HLA, vertical long axis—VLA, and left ventricular outflow tract—LVOT cines both sagittal and coronal) and a complete short axis (SA) stack of balanced steady state free precession (bSSFP) cines, covering the left ventricle (LV) and right ventricle (RV) are acquired [14]. For all measured cardiac parameters, a CMR reference standard has been created for the UK Biobank using 5,065 CMR scans as previously described [15]. The manual analysis of CMR scans was performed across two core laboratories based in London and Oxford using cvi42 post-processing software (Version 5.1.1, Circle Cardiovascular Imaging Inc., Calgary, Canada).

### Handgrip strength measurement

Handgrip strength was measured at baseline and at the imaging visit using a Jamar J00105 hydraulic hand dynamometer. The participant was asked to squeeze the handle of the dynamometer as strongly as possible for three seconds. At both visits, one measurement was obtained from each hand.

### Statistical analysis and model development

The primary exposures of interest in our models were (i) Handgrip strength at baseline and (ii) change in handgrip strength between baseline and the imaging visit. Even though handgrip strength was measured in both hands, we limited the analysis to the highest measurement at each visit because of very high correlations between handgrip strength measurements.

The outcomes of interest were derived from the manually verified CMR results [15] and included: (i) left ventricular ejection fraction (LVEF), (ii) left ventricular end-diastolic volume (LVEDV), (iii) left ventricular end-systolic volume (LVESV), (iv) left ventricular stroke volume (LVSV), (v) left ventricular mass (LVM), and (vi) left ventricular mass to volume ratio (LVMVR), a CMR measure of cardiac adaptation previously described [12, 15].

In all statistical models, we adjusted for: (i) baseline demographics, (ii) cardiac risk factors, (iii) drivers of muscle mass, and (iv) physical activity level, measured in metabolic equivalent of task (MET) minutes [16], mean centered as detailed in Table 1 (full details are available in the supporting information [S1 Methods and S1 Table]). All potential confounders were selected a priori.

**Table 1:** Participant characteristics according to baseline handgrip strength.

	Entire sample (N = 4,654)	Missing, N (%)
Handgrip strength, kg		
Baseline	34.9 (11.2)	8 (0.2)
Change	-4.1 (6.1)	32 (0.7)
*Cardiac parameters*		
LVEF, %	59.5 (6.3)	0 (0)
LVEDV, ml	143.4 (33.9)	0 (0)
LVESV, ml	58.7 (19.6)	0 (0)
LVSV, ml	84.6 (19.3)	0 (0)
LVM, g	89.1 (24.6)	0 (0)
LVMVR, g/ml	0.626 (0.120)	0 (0)
*Demographics*		
Age, years	55.8 (7.6)	0 (0)
Time baseline visit—imaging, years	6.3 (1.0)	0 (0)
Male sex	2163 (46.5)	0 (0)
Caucasian	4382 (94.4)	13 (0.3)
Standing height, cm	169.4 (9.2)	4 (0.1)
Weight, kg	76.7 (14.9)	59 (1.3)
Body fat, %	30.5 (8.2)	61 (1.3)
Waist circumference, cm	88 (12.6)	3 (0.1)
Hip circumference, cm	102.2 (8.2)	3 (0.1)
Townsend score	-1.9 (2.7)	2 (0)
Household income		423 (9.1)
<18k £ / year	604 (14.3)	
18k–31k £ / year	1033 (24.4)	
31k–52k £ / year	1281 (30.3)	
52k–100k £ / year	1051 (24.8)	
>100k £ / year	262 (6.2)	
Advanced degree	2759 (59.5)	15 (0.3)
*Cardiac risk factors*		
Hypertension	982 (21.1)	0 (0)
SBP, mmHg	135.3 (17.6)	94 (2.0)
DBP, mmHg	81.5 (9.9)	94 (2.0)
Diabetes mellitus	120 (2.6)	0 (0)
Dyslipidemia	529 (11.4)	0 (0)
Positive family history	3316 (71.3)	12 (0.3)
Tobacco use		0 (0)
Never	2759 (59.4)	
Former (light)	514 (11.1)	
Former (heavy)	1063 (22.9)	
Current (light)	116 (2.5)	
Current (heavy)	190 (4.1)	
*Drivers of muscle mass*		
Alcohol use		4 (0.1)
Never	262 (5.6)	
On special occasions	395 (8.5)	
One to three times / month	504 (10.8)	
Once or twice / week	1186 (25.5)	
Three or four times / week	1241 (26.7)	
Daily	1062 (22.8)	
Cancer	298 (6.4)	9 (0.2)
Physical activity level		
Total physical activity (MET minutes)	2796.6 (3512.2)	740 (15.9)
Days/week walked >10min	5.2 (2.0)	29 (0.6)
Duration of walks, min	55.3 (67.6)	447 (9.6)
Days / week moderate activity	3.5 (2.3)	120 (2.6)
Duration of activity, min	51.8 (66.7)	426 (9.2)
Days / week vigorous activity	1.9 (1.9)	95 (2)
Duration of activity, min	28.4 (40.9)	273 (5.9)

Numbers are mean (SD) or number (%), unless otherwise stated. Tertile sizes may vary because of ties in the data.

LVEF, left ventricular ejection fraction; LVEDV, left ventricular end-diastolic volume; LVESV, left ventricular end-systolic volume; LVSV, left ventricular stroke volume; LVM, left ventricular mass; LVMVR, left ventricular mass to volume ratio; SBP, systolic blood pressure; DBP, diastolic blood pressure; MET minutes, metabolic equivalent of task minutes.

We used separate multivariable linear regression models to investigate the relationship between baseline handgrip strength and change in handgrip strength and each of the cardiac outcome parameters and expressed the results per standard deviation (SD) increase in handgrip strength / change in handgrip strength (mean centered). To evaluate whether the observed associations differed by age, sex and physical activity level (MET minutes), we included product terms between handgrip strength and each of these potential modifiers.

Although missingness was generally low, we used multiple imputation by chained equations (MICE) to generate 10 complete datasets [17] (full details are available in the supporting information [S1 Methods]). In brief, we used predictive mean matching with three nearest neighbors for continuous variables, logistic regression for binary variables, and multinomial logistic regression for categorical variables. Rubin’s rule [18] was used to pool estimates and standard errors of the beta coefficients as well as predictions [17, 19]. Chi-square values of likelihood ratio test were pooled as recommended by Meng and Rubin [20]. Figures shown are for a single imputed data set in order to be able to use Stata’s commands ‘adjustrcspline’, ‘margins’, and ‘marginsplot’. Confidence intervals pooled across all ten imputation sets were less than 1% wider than those presented in the figures.

We conducted the following sensitivity analyses: (i) an analysis with the restricted-cubic-spline-transformed exposures, investigating the relationship between handgrip strength and the cardiac outcome parameters We used restricted cubic spline transformations with five knots and knot locations as recommended by Harrell [21] if a non-linear relationship was observed between handgrip strength or change in handgrip strength and any outcome conditional on the covariates. Non-linearity was defined as a p-value of <0.05 of a likelihood-ratio (LR) test comparing the model with the transformed predictor to the model including only the linear term; (ii) an analysis excluding hypertension, systolic blood pressure, diastolic blood pressure, and diabetes mellitus given the possibility that these might be important mediators of the association between handgrip strength on the cardiac outcomes rather than confounders [22, 23]; and (iii) an analysis of participants with complete data. We used Stata v.14.1 (StataCorp, College Station, Texas, USA) for all statistical analyses.

## Results

### Sample characteristics

**Fig 1:**
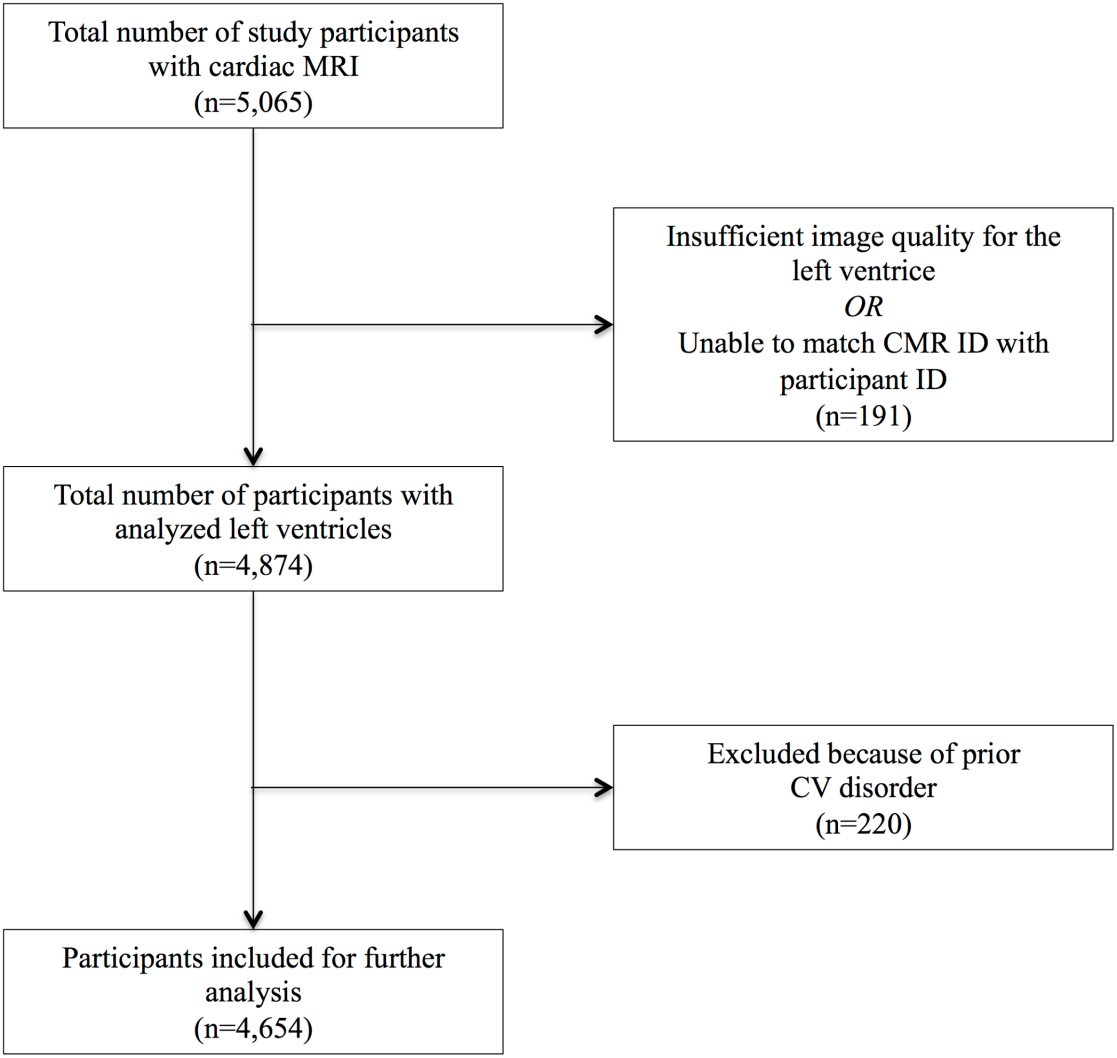
Study population.

By April 2017, cardiac parameters had been measured in 5,065 individuals. Of those, the left ventricular function was analyzed in 4,874 participants. After exclusion of 220 individuals with prior cardiovascular disorders, 4,654 individuals (46.5% male, mean 55.8 years of age) were included in our study (Fig 1). Mean baseline handgrip strength was 34.9 kg. Individuals with higher levels of handgrip strength were younger, taller, heavier, and had a higher household income. However, other demographic characteristics were similar among handgrip strength strata (Table 1). The amount of missing data ranged from 0% to 9.6% (duration of moderate physical activity / week).

### Association of baseline handgrip strength with cardiac structure and function

Table 2 shows the associations between baseline handgrip strength and cardiac outcome parameters after adjustment for all covariates. Higher baseline handgrip strength was significantly associated with higher LVEDV (difference per SD increase in handgrip strength 1.3ml, 95% CI 0.1–2.4; p = 0.036) and LVSV (1.0ml, 0.3–1.7; p = 0.007). There was a significant negative association with LVM (-1.0g, -1.8 –-0.3; p = 0.007) and LVMVR (-0.012g/ml, -0.18 –-0.007; p<0.001). No clear association was found between handgrip strength and LVEF or LVESV.

**Table 2:** Association between baseline handgrip strength and cardiac structure and function, adjusted for all covariates.

Adjusted model	Difference per SD increase in baseline handgrip strength
LVEF	0.17% (-0.13–0.48); p = 0.265
LVEDV	1.25ml (0.08–2.43); p = 0.036
LVESV	0.23ml (-0.55–1.00); p = 0.565
LVSV	1.01ml (0.28–1.74); p = 0.007
LVM	-1.03g (-1.79 –-0.28); p = 0.007
LVMVR	-0.012g/ml (-0.018 –-0.007); p<0.001

Numbers are difference (95% CI).

All estimates are adjusted for age, sex, ethnicity, time between baseline and imaging, height, weight, percent body fat, waist circumference, hip circumference, Townsend score, household income, educational attainment, hypertension, systolic blood pressure, diastolic blood pressure, diabetes mellitus, dyslipidemia, family history for cardiovascular disease, smoking, alcohol consumption, cancer, and physical activity level.

SD, standard deviation; LVEF, left ventricular ejection fraction; LVEDV, left ventricular end-diastolic volume; LVESV, left ventricular end-systolic volume; LVSV, left ventricular stroke volume; LVM, left ventricular mass; LVMVR, left ventricular mass to volume ratio.

**Fig 2:**
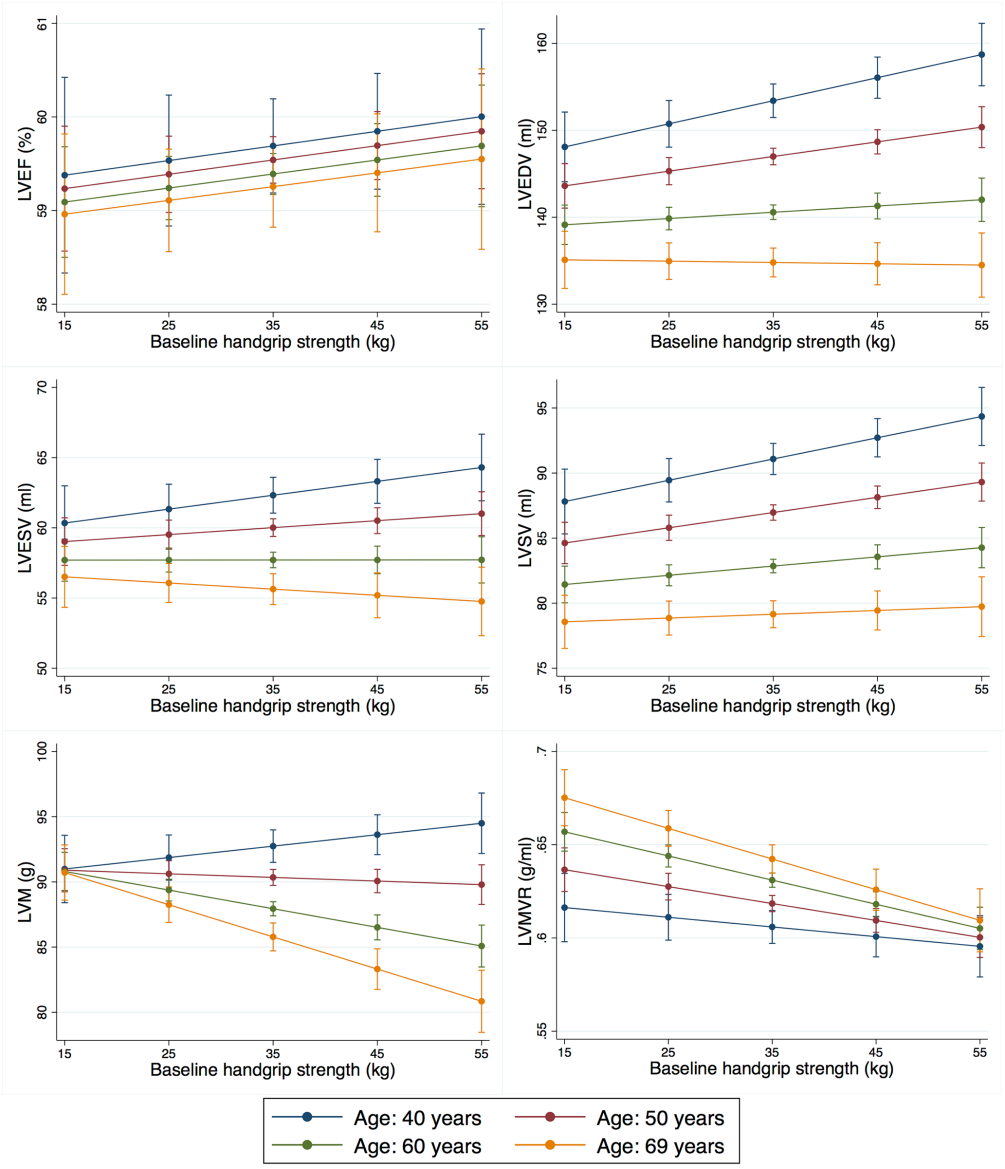
Association between baseline handgrip strength and cardiac structure and function by age, adjusted for all covariates. The figure shows the association between baseline handgrip strength and the cardiac outcome parameters by age after adjustment for all covariates. Intervals of baseline handgrip strength were chosen to closely represent one standard deviation with a mean at approximately 35 kg. Error bars represent 95% CI. Baseline handgrip strength has a stronger association with LVEDV and LVSV among younger individuals and a stronger association with LVM and LVMVR among older individuals. HGS, handgrip strength; LVEF, left ventricular ejection fraction; LVEDV, left ventricular end-diastolic volume; LVESV, left ventricular end-systolic volume; LVSV, left ventricular stroke volume; LVM, left ventricular mass; LVMVR, left ventricular mass to volume ratio.

**Fig 3:**
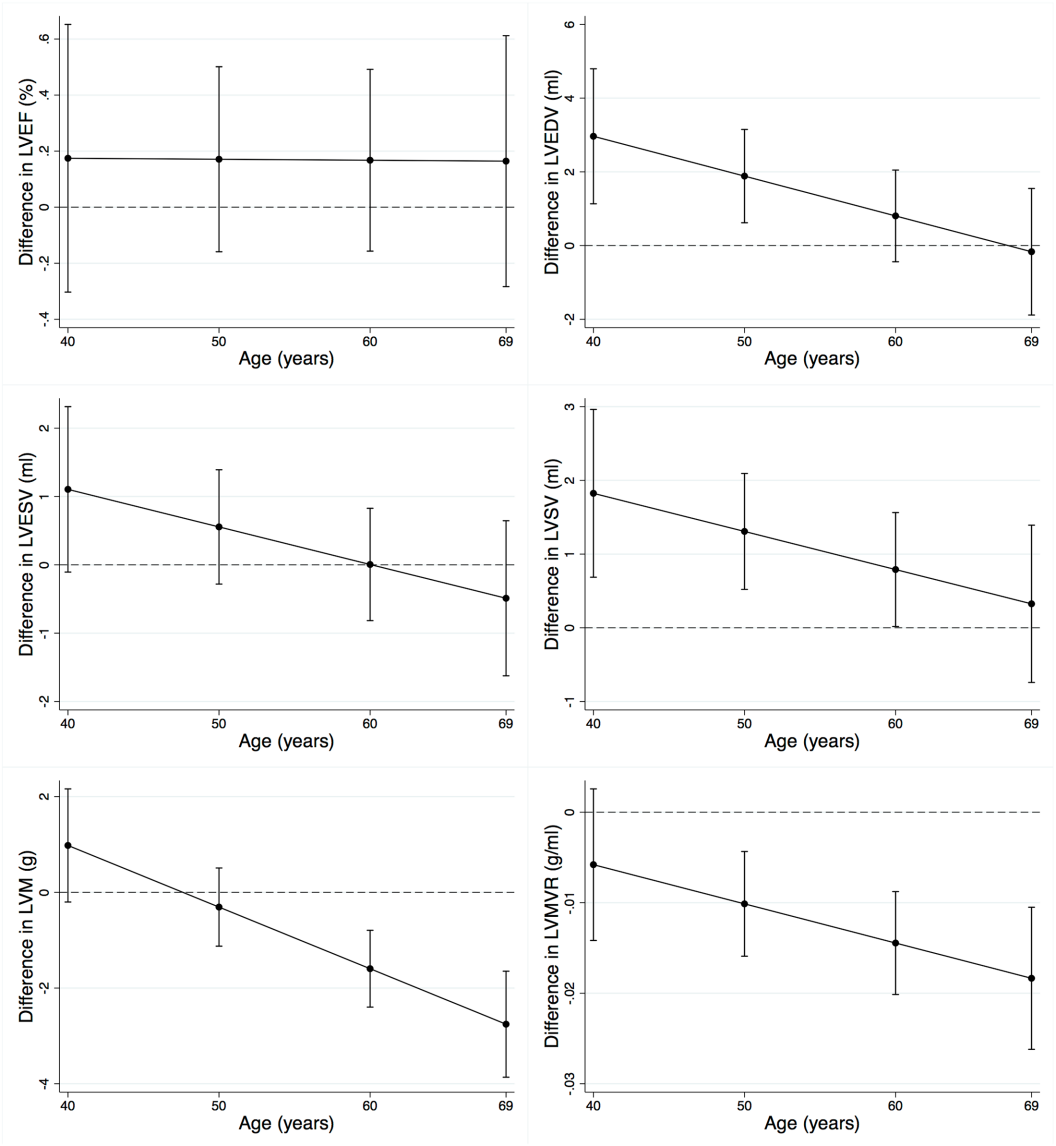
Association between baseline handgrip strength and the difference in cardiac structure and function by age, adjusted for all covariates. The figure shows the difference in each cardiac outcome parameter per one standard deviation increase in baseline handgrip strength by age after adjustment for all covariates. Error bars represent 95% CI. Baseline handgrip strength has a stronger association with LVEDV and LVSV among younger individuals and a stronger association with LVM and LVMVR among older individuals. LVEF, left ventricular ejection fraction; LVEDV, left ventricular end-diastolic volume; LVESV, left ventricular end-systolic volume; LVSV, left ventricular stroke volume; LVM, left ventricular mass; LVMVR, left ventricular mass to volume ratio.

There was evidence that the association between baseline handgrip strength and LVEDV, LVSV, LVM, and LVMVR varied by age, but not between men and women or across levels of physical activity. The association with LVEDV and LVSV was strongest among younger individuals, while the association with LVM and LVMVR was strongest among older individuals. Among 40 year olds, higher levels of handgrip strength at baseline were associated with higher LVEDV (2.9ml, 1.1–4.8; p = 0.002) and LVSV (1.8ml, 0.7–3.0; p = 0.002). These associations decreased with age. There was no clear association between baseline handgrip strength and LVM or LVMVR among 40 year olds. However, these associations increased with age. Among 69 year olds, higher levels of handgrip strength at baseline were associated with lower LVM (-2.8g, -3.9 –-1.7; p<0.001) and LVMVR (-0.018g/ml, -0.026 –-0.011; p<0.001) (Figs 2 and 3).

### Association of change in handgrip strength with cardiac structure and function

No association was found between change in handgrip strength and any of the CMR-based measures of cardiac structure and function after adjustment for the covariates, nor was there evidence that results varied by age, sex or physical activity (data not shown).

### Sensitivity analyses

There was no consistent evidence that the observed associations between handgrip strength and the CMR-based measures of cardiac structure and function were non-linear (S1 Fig). Neither the results from analyses that did not adjust for hypertension, systolic blood pressure, diastolic blood pressure and diabetes, nor the results from analyses of participants with complete data were materially different from the primary analyses.

## Discussion

The association between handgrip strength, a measure of muscular fitness, and measures of cardiac structure and function was not previously known. This is the first study to show that higher levels of handgrip strength are associated with higher LVEDV and LVSV, and lower LVM and LVMVR. The association with LVEDV and LVSV decreased with age while the association with LVM and LVMVR increased with age. These findings advance our understanding of the pathophysiologic processes that may mediate the association between handgrip strength and cardiovascular incidence and mortality.

Two large Swedish studies [6, 7] showed lower cardiovascular disease incidence and mortality among male adolescents with higher levels of handgrip strength. The PURE study [3] recently demonstrated a similar association across people of a wide age range and diverse economic and sociocultural backgrounds. Those studies did not, however, investigate possible mechanisms responsible for the observed associations.

Lower handgrip strength among younger individuals was associated with a pattern resembling concentric remodeling, a process characterized by a lower LVEDV, no difference in LVM, and higher LVMVR [12]. Among older individuals, lower handgrip strength was associated with a pattern resembling concentric hypertrophy, a process characterized by higher LVM, no difference in LVEDV, and higher LVMVR [12]. It is not surprising that we did not see an association between handgrip strength and LVEF, since such changes are only expected to occur in LV decompensation. LV hypertrophy and concentric remodeling have been associated with a marked increase in adverse CVD events in the general population [24] as well as outcome events in patients with heart failure [25], which could link handgrip strength to CVD incidence.

Our results were not materially altered in models that did not adjust for other cardiac risk factors such as hypertension and diabetes mellitus suggesting that these risk factors do not strongly mediate the association between muscular fitness as assessed by handgrip strength and the CMR-based measures of structure and function. This is in line with the PURE study [3] that showed that the association between handgrip strength and cardiovascular disease incidence and mortality persisted after adjustment for these risk factors.

The absence of an association between change in handgrip strength and any cardiac outcome parameter was unexpected. However, several features of the study design preclude a strong interpretation of those results: CMR was performed only once at the end of the study, but not at baseline. The associations found for baseline handgrip strength therefore likely represent the relationship between handgrip strength and the CMR-based measures over a time period that far exceeds the study period.

Our study has several notable strengths including a large population-based study sample; standardized data collection protocols as part of the UK Biobank prospective cohort study; CMR-based measures of cardiac structure and function measured with a consistent research protocol; and robust results across a wide range of sensitivity analyses.

Like all observational studies, our study also had some limitations. Even though we adjusted for many potential confounders, residual confounding cannot be excluded. Furthermore, CMR was performed only once at the end of the study and therefore reverse causation cannot be excluded. However, it seems unlikely given the consistency of the results with previous studies investigating clinical outcomes. Finally, as a population based study the UK Biobank was planned without administration of contrast agents and therefore gadolinium / relaxometry imaging was not available.

In conclusion, better handgrip strength was associated with the CMR-based measures of cardiac structure and function that are indicative of less cardiac hypertrophy and remodeling. Those characteristics are known to be negatively associated with CVD incidence. Handgrip grip strength might, thus, allow early identification of individuals at risk for development of CVD. Focused surveillance and intervention may improve outcomes, but further research is necessary to assess whether fitness training can reduce cardiac remodeling and prevent cardiovascular events.

## Supporting information

S1 Methods (DOC)

S1 Table Participant characteristics according to baseline handgrip strength. (DOCX)

S1 Fig Association between transformed baseline handgrip strength and cardiac structure and function. (DOCX)
