# Supplementary material for: Prospective association between handgrip strength and cardiac structure and function in UK adults
Source: PLoS One. 2018 Mar 14;13(3):e0193124. doi: 10.1371/journal.pone.0193124 (PMC5851546; doi:10.1371/journal.pone.0193124)
Supplement: S1 Methods — (DOC) [file pone.0193124.s001.doc]

**S1 Methods**

**Exclusion criteria**

We excluded participants with any history of cardiovascular conditions including angina, heart attack/myocardial infarction, heart failure/pulmonary edema, heart arrhythmia, cardiomyopathy, heart valve problem/heart murmur, mitral stenosis, aortic stenosis, mitral valve disease, mitral regurgitation/incompetence, aortic valve disease, aortic regurgitation/incompetence, hypertrophic cardiomyopathy (HCM/HOCM), pericardial problem, atrial fibrillation, stroke, ischemic stroke, transient ischemic attack or peripheral vascular disease.

**Statistical Analysis and Model Development**

**Adjustment for confounders**

In all statistical models, we adjusted for: (i) baseline demographics – age, centered at 40 years; time between baseline assessment and the imaging visit; sex; ethnicity, dichotomized into Caucasian and non-Caucasian; standing height; weight; percent body fat; waist circumference; hip circumference; Townsend Area Deprivation Score; household income, categorized into <18,000 £ / year, 18,000 – 31,000 £ / year, 31,000 – 52,000 £ / year, 52,000 – 100,000 £ / year, and > 100,000 £ / year; educational attainment, dichotomized into college/university degree or professional qualifications and no college/university degree or professional qualifications, (ii) cardiac risk factors - hypertension, based on self-reported medical conditions as well as prescribed medications; systolic blood pressure; diastolic blood pressure; dyslipidemia, based on prescribed medications; diabetes mellitus, based on self-reported medical conditions, prescribed medications, and diagnoses by a physician; positive family history for heart disease, stroke, or hypertension; any tobacco smoking, categorized into never, former (light [occasionally]), former (heavy [on most or all days]), current (light [occasionally]), and current (heavy [on most or all days]), (iii) drivers of muscle mass - alcohol use, categorized into never, on special occasions, one to three times per month, once or twice per week, three or four times per week, and daily; self-reported cancer, and (iv) physical activity level, measured in metabolic equivalent of task (MET) minutes [1], mean centered. All potential confounders were selected a priori.

**Multiple imputation by chained equations (MICE)**

We used multiple imputation by chained equations (MICE) to generate 10 complete datasets [2]. We used predictive mean matching with three nearest neighbors for continuous variables, logistic regression for binary variables, and multinomial logistic regression for categorical variables. We specified an imputation model that was congenial to all the analysis models. Splines and interaction terms, except those that included total physical activity (MET minutes), were included as just another variable. The composite score for physical activity (MET minutes) was not used in the imputation model based on simulations estimating the mean absolute error (data not shown). Instead, the six separate variables (Days / week walked > 10 min, duration of walks, days / week with moderate activity, duration of moderate activity, days / week with vigorous activity, and duration of vigorous activity) were imputed and the composite score as well as interaction terms that included total physical activity were then recalculated for use in the final analysis model.

Calibration and centering of variables (except of activity) were performed before the imputation. Burn-in was set to 20 repetitions and convergence was confirmed by visual examination of trace plots. Rubin’s rule [3] was used to pool estimates and standard errors of the beta coefficients as well as predictions [2, 4]. Chi-square values of likelihood ratio test were pooled as recommended by Meng and Rubin [5]. Figures shown are for a single imputed data set in order to be able to use Stata’s commands ‘adjustrcspline’, ‘margins’, and ‘marginsplot’. Confidence intervals pooled across all ten imputation sets were less than 1% wider than those presented in the figures.

**References**
