# Supplementary material for: Prospective association between handgrip strength and cardiac structure and function in UK adults
Source: PLoS One. 2018 Mar 14;13(3):e0193124. doi: 10.1371/journal.pone.0193124 (PMC5851546; doi:10.1371/journal.pone.0193124)
Supplement: S1 Table — (DOCX) [file pone.0193124.s002.docx]

**S1 Table.** Participant characteristics according to baseline handgrip strength.

|  | Entire sample  (N=4,654) | Missing,  N (%) | Women | | | Men | | |
| --- | --- | --- | --- | --- | --- | --- | --- | --- |
|  |  |  | First  Tertile  (N=888) | Second Tertile  (N=959) | Third Tertile  (N=635) | First  Tertile  (N=787) | Second Tertile  (N=775) | Third Tertile  (N=589) |
| Handgrip strength, kg |  |  |  |  |  |  |  |  |
| Baseline | 34.9 (11.2) | 8 (0.2) | 20.5 (3.6) | 28 (1.7) | 35.1 (3.5) | 35.2 (4.9) | 44.7 (2.3) | 54.1 (4.4) |
| Change | -4.1 (6.1) | 32 (0.7) | -0.4 (5.1) | -4.2 (4.2) | -7.1 (5.1) | -1.5 (6.4) | -5.4 (6) | -8 (6.3) |
|  |  |  |  |  |  |  |  |  |
| *Cardiac parameters* |  |  |  |  |  |  |  |  |
| LVEF, % | 59.5 (6.3) | 0 (0) | 60.9 (5.9) | 60.5 (6.1) | 60.5 (6.2) | 58 (6.4) | 58.3 (6.6) | 57.8 (6.1) |
| LVEDV, ml | 143.4 (33.9) | 0 (0) | 119.8 (20.3) | 125.7 (22) | 134.3 (25.4) | 156.3 (31.6) | 163.6 (33.7) | 173.4 (31.4) |
| LVESV, ml | 58.7 (19.6) | 0 (0) | 47.1 (11.6) | 49.8 (13.1) | 53.4 (16) | 66.1 (19.2) | 68.8 (21.8) | 73.5 (18.9) |
| LVSV, ml | 84.6 (19.3) | 0 (0) | 72.7 (13.2) | 75.9 (14.2) | 80.9 (15.5) | 90.3 (18.5) | 94.8 (19.4) | 99.9 (19.2) |
| LVM, g | 89.1 (24.6) | 0 (0) | 71.9 (13.9) | 73.8 (14.4) | 77.3 (16.4) | 103.1 (21.5) | 106.3 (20.9) | 111.4 (23.1) |
| LVMVR, g/ml | 0.626  (0.120) | 0 (0) | 0.608 (0.112) | 0.594  (0.106) | 0.582 (0.099) | 0.670 (0.125) | 0.662 (0.125) | 0.650 (0.120) |
|  |  |  |  |  |  |  |  |  |
| *Demographics* |  |  |  |  |  |  |  |  |
| Age, years | 55.8 (7.6) | 0 (0) | 57.7 (6.9) | 55.3 (7.4) | 51.7 (7.3) | 58.6 (7.3) | 56.4 (7.4) | 53.6 (7.4) |
| Time baseline visit -  imaging, years | 6.3 (1.0) | 0 (0) | 6.1 (1.0) | 6.3 (1.0) | 6.6 (0.9) | 6.1 (1.0) | 6.3 (1.0) | 6.5 (1.0) |
| Male sex | 2163 (46.5) | 0 (0) |  |  |  |  |  |  |
| Caucasian | 4382 (94.4) | 13 (0.3) | 838 (94.4) | 906 (94.5) | 606 (95.4) | 740 (94.0) | 731 (94.3) | 553 (93.9) |
| Standing height, cm | 169.4 (9.2) | 4 (0.1) | 160.9 (5.9) | 163.2 (6.1) | 166.2 (5.8) | 174 (6.3) | 176.9 (6.1) | 179.3 (6) |
| Weight, kg | 76.7 (14.9) | 59 (1.3) | 68 (11.9) | 70 (12.5) | 71.7 (13.1) | 81.9 (13.1) | 84.4 (12.6) | 88.6 (13.2) |
| Body fat, % | 30.5 (8.2) | 61 (1.3) | 36.3 (6.4) | 35.7 (6.4) | 34.7 (7) | 25.2 (5.4) | 24.4 (5.4) | 23.8 (5.2) |
| Waist circumference, cm | 88 (12.6) | 3 (0.1) | 82.5 (11.4) | 81.9 (11.3) | 81.8 (11.2) | 94.7 (10.7) | 94.5 (10.6) | 95.1 (10.2) |
| Hip circumference, cm | 102.2 (8.2) | 3 (0.1) | 101.1 (9.1) | 102.2 (9.1) | 102.5 (9.2) | 101.8 (7) | 102.5 (7) | 104.1 (6.7) |
| Townsend score | -1.9 (2.7) | 2 (0) | -1.9 (2.7) | -1.9 (2.7) | -1.9 (2.7) | -1.8 (2.8) | -1.9 (2.8) | -2.1 (2.6) |
| Household income |  | 423 (9.1) |  |  |  |  |  |  |
| <18k £ / year | 604 (14.3) |  | 168 (22.0) | 138 (16.4) | 64 (10.9) | 114 (15.5) | 81 (11.0) | 38 (6.8) |
| 18k – 31k £ / year | 1033 (24.4) |  | 240 (31.5) | 219 (26.0) | 119 (20.3) | 174 (23.6) | 176 (23.9) | 104 (18.7) |
| 31k – 52k £ / year | 1281 (30.3) |  | 197 (25.8) | 255 (30.3) | 189 (32.2) | 215 (29.2) | 237 (32.2) | 185 (33.21) |
| 52k – 100k £ / year | 1051 (24.8) |  | 130 (17.0) | 191 (22.7) | 169 (28.8) | 182 (24.7) | 199 (27.0) | 178 (32.0) |
| >100k £ / year | 262 (6.2) |  | 28 (3.7) | 40 (4.7) | 46 (7.8) | 52 (7.1) | 44 (6.0) | 52 (9.3) |
| Advanced degree | 2759 (59.5) | 15 (0.3) | 463 (52.2) | 549 (57.3) | 406 (64) | 487 (61.8) | 475 (61.3) | 373 (63.3) |
|  |  |  |  |  |  |  |  |  |
| *Cardiac risk factors* |  |  |  |  |  |  |  |  |
| Hypertension | 982 (21.1) | 0 (0) | 182 (20.4) | 176 (18.4) | 89 (14) | 185 (23.4) | 206 (26.5) | 143 (24.2) |
| SBP, mmHg | 135.3 (17.6) | 94 (2.0) | 132.9 (18.3) | 131.8 (18.5) | 130.5 (17.3) | 139.1 (16) | 139.7 (16.3) | 139.1 (15.9) |
| DBP, mmHg | 81.5 (9.9) | 94 (2.0) | 79.8 (10.0) | 79.6 (9.7) | 79.1 (9.5) | 83.0 (9.1) | 83.9 (9.6) | 84.6 (9.8) |
| Diabetes mellitus | 120 (2.6) | 0 (0) | 15 (1.7) | 26 (2.7) | 10 (1.6) | 27 (3.4) | 25 (3.2) | 16 (2.7) |
| Dyslipidemia | 529 (11.4) | 0 (0) | 77 (8.6) | 90 (9.4) | 29 (4.6) | 130 (16.4) | 121 (15.6) | 81 (13.7) |
| Positive family history | 3316 (71.3) | 12 (0.3) | 661 (74.2) | 701 (73.1) | 468 (73.5) | 550 (69.4) | 535 (68.9) | 395 (66.9) |
| Tobacco use |  | 0 (0) |  |  |  |  |  |  |
| Never | 2759 (59.4) |  | 542 (61.1) | 617 (64.5) | 391 (61.6) | 424 (53.7) | 445 (57.3) | 336 (57) |
| Former (light) | 514 (11.1) |  | 102 (11.5) | 114 (11.9) | 73 (11.5) | 85 (10.8) | 71 (9.1) | 68 (11.5) |
| Former (heavy) | 1063 (22.9) |  | 198 (22.3) | 176 (18.4) | 123 (19.4) | 228 (28.9) | 198 (25.5) | 137 (23.3) |
| Current (light) | 116 (2.5) |  | 14 (1.6) | 16 (1.7) | 14 (2.2) | 21 (2.7) | 29 (3.7) | 22 (3.7) |
| Current (heavy) | 190 (4.1) |  | 31 (3.5) | 33 (3.5) | 34 (5.4) | 32 (4.1) | 34 (4.4) | 26 (4.4) |
|  |  |  |  |  |  |  |  |  |
| *Drivers of muscle mass* |  |  |  |  |  |  |  |  |
| Alcohol use |  | 4 (0.1) |  |  |  |  |  |  |
| Never | 262 (5.6) |  | 62 (7) | 61 (6.4) | 31 (4.9) | 43 (5.4) | 36 (4.6) | 28 (4.7) |
| On special occasions | 395 (8.5) |  | 118 (13.3) | 109 (11.4) | 50 (7.9) | 47 (5.9) | 42 (5.4) | 28 (4.7) |
| One to three times / month | 504 (10.8) |  | 122 (13.7) | 128 (13.3) | 81 (12.7) | 66 (8.3) | 62 (8) | 44 (7.5) |
| Once or twice / week | 1186 (25.5) |  | 210 (23.6) | 252 (26.3) | 176 (27.7) | 196 (24.8) | 193 (24.9) | 157 (26.6) |
| Three or four times / week | 1241 (26.7) |  | 216 (24.3) | 230 (24) | 161 (25.3) | 222 (28.1) | 235 (30.3) | 176 (29.8) |
| Daily | 1062 (22.8) |  | 162 (18.2) | 179 (18.7) | 137 (21.5) | 217 (27.4) | 208 (26.8) | 157 (26.6) |
| Cancer | 298 (6.4) | 9 (0.2) | 76 (8.5) | 68 (7.1) | 33 (5.2) | 46 (5.8) | 45 (5.8) | 30 (5.1) |
|  |  |  |  |  |  |  |  |  |
| Physical activity level |  |  |  |  |  |  |  |  |
| Total physical activity  (MET minutes) | 2796.6 (3512.2) | 740  (15.9) | 2699.1 (3254.7) | 2743.0 (3155.7) | 2914.2 (3445.2) | 2704.5 (3477.0) | 2906.6 (3920.1) | 2846.7 (3868.3) |
| Days/week walked >10min | 5.2 (2.0) | 29 (0.6) | 5.2 (2) | 5.4 (1.9) | 5.3 (2) | 5.1 (2) | 5.1 (2) | 5.1 (2) |
| Duration of walks, min | 55.3 (67.6) | 447 (9.6) | 54.9 (63.3) | 55.0 (65.6) | 62.4 (78.9) | 54.4 (66.9) | 54.5 (68.4) | 50.9 (63.3) |
| Days / week moderate activity | 3.5 (2.3) | 120 (2.6) | 3.6 (2.3) | 3.5 (2.3) | 3.5 (2.2) | 3.4 (2.3) | 3.4 (2.3) | 3.4 (2.3) |
| Duration of activity, min | 51.8 (66.7) | 426 (9.2) | 52.9 (61.9) | 51.7 (62.3) | 50.3 (64.9) | 50.3 (70.1) | 54.6 (74.0) | 49.2 (66.9) |
| Days / week vigorous activity | 1.9 (1.9) | 95 (2) | 1.5 (1.8) | 1.7 (1.8) | 1.8 (1.7) | 1.9 (2) | 2.1 (2) | 2.1 (1.9) |
| Duration of activity, min | 28.4 (40.9) | 273 (5.9) | 22.7 (35.2) | 27.0 (37.7) | 30.3 (40.1) | 27.3 (40.5) | 32.0 (46.1) | 33.6 (46.5) |

Numbers are mean (SD) or number (%), unless otherwise stated. Tertile sizes may vary because of ties in the data.

LVEF, left ventricular ejection fraction; LVEDV, left ventricular end-diastolic volume; LVESV, left ventricular end-systolic volume; LVSV, left ventricular stroke volume; LVM, left ventricular mass; LVMVR, left ventricular mass to volume ratio; SBP, systolic blood pressure; DBP, diastolic blood pressure; MET minutes, metabolic equivalent of task minutes.
