# Supplementary material for: Prospective association between handgrip strength and cardiac structure and function in UK adults
Source: PLoS One. 2018 Mar 14;13(3):e0193124. doi: 10.1371/journal.pone.0193124 (PMC5851546; doi:10.1371/journal.pone.0193124)
Supplement: S1 Fig — (DOCX) [file pone.0193124.s003.docx]

**S1 Figure.** Association between transformed baseline handgrip strength and cardiac structure and function.

A restricted cubic spline transformation with 5 knots was used to test for non-linearity of the relationship between baseline handgrip strength and the cardiac outcome parameters conditional on all covariates. All covariates are set to their mean value. The shaded area represents the 95% confidence interval.

None of the plots shows a biologically plausible non-linear relationship of baseline handgrip strength with any cardiac outcome parameter. Slight deviations from linearity for very low values of handgrip strength may be in the setting of medical conditions that could preclude adequate measurement.

LVEF, left ventricular ejection fraction; LVEDV, left ventricular end-diastolic volume; LVESV, left ventricular end-systolic volume; LVSV, left ventricular stroke volume; LVM, left ventricular mass; LVMVR, left ventricular mass to volume ratio.
